# Methane emission, intake, digestibility, performance and blood metabolites in sheep supplemented with cupuassu and tucuma cake in the eastern Amazon

**DOI:** 10.3389/fvets.2023.1106619

**Published:** 2023-04-25

**Authors:** Juliana Cristina de Castro Budel, Vinicius Costa Gomes de Castro, Shirley Motta de Souza, Benjamin de Souza Nahúm, Antônio Vinicius Corrêa Barbosa, Laurena Silva Rodrigues, Alexandre Berndt, Luciana Navajas Rennó, Eziquiel de Morais, Jamile Andrea Rodrigues da Silva, Thomaz Cyro Guimarães de Carvalho Rodrigues, André Guimarães Maciel e Silva, José de Brito Lourenço-Junior

**Affiliations:** ^1^Institute of Veterinary Medicine, Postgraduate Program in Animal Science, Federal University of Pará, Abaetetuba, Pará, Brazil; ^2^Institute of Veterinary Medicine, Postgraduate Program in Animal Science, Federal Rural University of the Amazon, Belém, Pará, Brazil; ^3^Department of Animal Science, Federal University of Viçosa, Minas Gerais, Brazil; ^4^Embrapa Eastern Amazon, Belém, Pará, Brazil; ^5^Statistics Department, University Federal Rural of the Amazon, Belém, Pará, Brazil; ^6^Embrapa Southeast Livestock, São Carlos, São Paulo, Brazil; ^7^Department of Animal Science, Federal Institute of Pará, Castanhal, Brazil; ^8^Institute of Veterinary Medicine, Postgraduate Program in Animal Science, Federal University of Pará, Belém, Pará, Brazil; ^9^Institute of Veterinary Medicine, Postgraduate Program in Animal Science, Federal University of Pará, Castanhal, Pará, Brazil

**Keywords:** agroindustry by-products, *Astrocaryum vulgare Mart*, feed efficiency, greenhouse gases, sustainability, *Theobroma grandiflorum*

## Abstract

The use of co-products as a feed supplement for ruminants makes livestock sustainable and optimizes the use of available areas and animal performance. Furthermore, when cakes are used, the residual fat composition can influence ruminal metabolism and methane (CH_4_) production. This study aimed to assess the effects of a diet containing cupuassu (CUP; *Theobroma grandiflorum*) and tucuma (TUC; *Astrocaryum vulgare Mart*.) cakes on intake, digestibility, serum metabolites, performance, and CH_4_ emissions in confined sheep in the Amazon. Approximately 28 animals, Dorper-Santa Inês, castrated, with an average initial live weight (ILW) of 35 ± 2.3 kg, were distributed in metabolic cages, in a completely randomized design, with four treatments and seven replications: (1) Control (C40), without the addition of Amazonian cake and with 40 g of ether extract (EE)/kg of dietary dry matter (DM); (2) CUP, the inclusion of the CUP cake and 70 g of EE/kg; (3) TUC, the inclusion of the TUC cake and 70 g of EE/kg; and (4) Control (C80), without the addition of Amazonian cake and with 80 g of EE/kg of dietary DM, with roughage to concentrate ratio of 40:60. The use of the TUC cake as a feed supplement reduced the intake of DM, crude protein (CP), and EE compared to the inclusion of the CUP cake (*p* < 0.05); however, it increased the intake of neutral detergent fiber (NDF) by 32% (*p* < 0.01). The highest averages of DM (732 g/kg) and CP (743 g/kg) digestibility were presented in C40, while the highest digestibility of NDF was presented in TUC (590 g/kg). Albumin levels stayed above and protein levels were below the reference values, and the C40 diet also obtained below results for cholesterol, triglycerides and High Density Lipoprotein (HDL) (*P* < 0.05). Sheep fed CUP (91 g) and TUC (45 g) had lower daily weight gains (DWGs) than those fed with diets without the inclusion of cakes (C40 = 119 g; C80 = 148 g), and feed efficiency (FE) was also lower in CUP (84) and TUC (60) diets than in C40 (119) and C80 (137) diets. CH_4_ emissions were lower in animals fed TUC (26 L/day) and higher in C40 (35 L/day); however, TUC resulted in higher CH_4_ emissions in grams/body live weight (BW) gain/day (353 g/BW/day) vs. 183 g/BW/day (C40), 157 g/BW/day (C80), and 221 g/BW/day (CUP). The supplementation with cakes did not improve intake, digestibility and performance, did not compromise blood metabolites and did not reduce the enteric CH4 emission in confined sheep in the Amazon; however, the use of CUP cake showed similar results to the control treatments and did not increase CH_4_ emissions, as occurred with the inclusion of TUC cake.

## 1. Introduction

With over 2 billion tons of agricultural residues accumulated worldwide, research on the management of this biomass is crucial for the preservation of the environment (1). Supplementation of ruminants with co-products has therefore been extensively studied and has shown promising results (2–4), including as a mitigator of daily methane (CH_4_) emissions (6–8).

The preservation and use of this material is also a relevant issue for the entire agri-food sector as it enables agribusiness to move toward circular economy models and sustainable production, promoting economic development with environmental and resource protection (9, 10).

Typical of the Brazilian Amazon, the fruits of cupuassu (CUP; *Theobroma grandiflorum Schum*) and tucuma (TUC; *Astrocaryum aculeatum*) have gone from extractive exploitation to domestic use (4, 11). This process has occurred due to increased national and international demand, mainly from the cosmetic and food industries (12, 13). This process increased the production of cakes, the co-product of raw material extraction, and increased the availability of human products.

The compositions of CUP and TUC cakes are 12.59–19.7 and 9.5–12.3 of protein; 51.4–54.7 and 55.4–69.3 of neutral detergent fiber (NDF); and 12.5–20.1 and 9.3–15.4 of ether extract (EE), respectively (2, 14). In addition, they have a differentiated fatty acid profile, with a predominance of saturated fats (56%) in CUP cakes and unsaturated fats (66%) in TUC cakes (15, 16). However, both have a higher percentage of oleic acid, which has been studied as a mitigator of enteric CH_4_ production (14, 17, 18). The inclusion of 1% of TUC oil, which is rich in oleic and palmitic acid, altered the pattern of ruminal fermentation and reduced CH_4_ emissions *in vitro* (14). The inclusion of up to 5% of CUP cake did not change with the intake, nutrient digestibility, and ingestive behavior of beef cattle; the inclusion of up to 40% of CUP cakes in diets for dairy cows is recommended; increased voluntary intake by sheep (19–22).

In addition to reducing inappropriate biomass disposal and improving productivity per area, its inclusion as an ingredient in ruminant diets can improve energy efficiency and reduce CH_4_ production. However, the effects of CUP and TUC cakes on consumption, digestibility, performance, and ovine methanogenesis *in vivo* still need to be studied.

We hypothesized that the inclusion of TUC and CUP cakes would reduce CH_4_ emissions without compromising production and biochemical parameters. Therefore, this study aimed to investigate the effects of Amazonian cakes on intake, digestibility, serum metabolites, performance, and CH_4_ emissions in feedlot sheep.

## 2. Materials and methods

This project was approved by the Ethics and Animal Welfare Committee of the Federal University of Pará, Faculty of Veterinary Medicine—Campus Castanhal, protocol number 8694141217.

### 2.1. Local, animal, and experimental design

The experiment was conducted at the experimental facility of the Federal Institute of Education, Science and Technology of Pará—IFPA (1°18′10.08^′′^ S, 47°56′56.10^′′^ W), Castanhal, Pará, with climate type Af, according to Köppen, average precipitation of 2,770 mm/year, and average annual temperature and relative air humidity of 26.8°C and 85%, respectively.

Approximately 28 castrated male Dorper-Santa Inês crossbred lambs with an average initial live weight (ILW) of 35 ± 2.3 kg and 9 ± 2 months of age were kept in metabolic cages (0.80 m × 1.20 m), equipped with both water and feed containers, arranged in a shed covered with tiles, and open on the sides for natural ventilation.

The experimental design was completely randomized, with four treatments (diets) and seven replicates, for 62 days, of which 7 days were for the animals' adaptation to diets, facilities, and the CH_4_ collection apparatus, and 55 days for dry matter intake (DMI) and performance evaluation. From days 50 to 54, collections were made to estimate intake, nutrient apparent digestibility, and enteric CH_4_ emissions. On day 55, blood was collected to determine serum metabolites.

### 2.2. Diets

Four diets were formulated with EE contents between 40 and 80 g/kg (dry matter (DM) basis). It consisted of 400 g/kg of forage (corn silage) and 600 g/kg of concentrate, with an estimated daily weight gain (DWG) of 200 g/animal (23). A high proportion of concentrate was adopted to allow a 7% fat diet and to obtain more significant results regarding the inclusion of cakes. For better evaluation, the work has a negative control, with approximately 4% EE (C40), and a positive control, with approximately 8% EE (C80). Both have the same EE source (soy). However, to reach 8% of EE (C80 diet), in addition to soybean meal, ground soybean, and soybean oil (commercial products from industrial processes) were added. Treatments with Amazonian cakes had ~7% of EE. The treatments were given as follows: (1) Control (C40), without Amazonian cake and with 40 g/kg of EE in total dry matter (TDM); (2) CUP, the inclusion of CUP cake (70 g/kg of EE); (3) TUC, the inclusion of TUC cake (70 g/kg of EE); and (4) Control (C80), without the addition of Amazonian cake but containing 80 g/kg of EE.

Amazonian cakes (CUP and TUC) were added to the diets to partially replace the corn and soybean meal. Both were the result of mechanical processing (in an Expeller press), carried out to remove oil from the seeds, by the company AmazonOil (Ananindeua, Pará), where they were acquired. The fruits, after being subjected to extraction in the hydraulic pen, generate oil, cake, and seed. Subsequently, the cake is separated from the seed and undergoes a new oil extraction, which makes it the least valuable material sold as a co-product. During the entire experimental period, the cakes were stored in 100-L barrels and corn silage was stored in 50-kg bags. The chemical composition of the diet ingredients is presented in Table 1.

**Table 1:** Chemical composition and fatty acids of the ingredients used to compose the experimental diets.

**Items**	**Ingredients**					
	**CS**	**GC**	**SM**	**GS**	**CUPcake**	**TUCcake**
**Nutrients, g/kg DM**						
Dry matter	228	885	957	906	955	956
Organic matter	939	986	933	950	940	978
Mineral matter	61	14	67	50	60	22
Crude protein	81	89	527	425	187	85
Ether extract	38	63	23	204	102	100
Neutral detergent fiber	504	147	248	264	317	690
NDFcp	488	131	231	246	300	676
ADFcp	249	16	115	154	301	395
Lignin	52	14	23	12	127	86
Non-fibrous carbohydrates	328	658	112	56	338	114
**Fatty acid composition, g/kg DM**						
Saturated fatty acids	257	286	315	63	560	275
Unsaturated fatty acids	748	714	685	937	448	666

CS, corn silage; GC, ground corn; SM, soybean meal; GS, ground soybean; CUPcake, cupuassu cake; TUCcake, tucuma cake; NDFcp, NDF corrected for ash and protein; ADFcp, corrected for ash and protein.

Diets were prepared and monitored every week in sufficient quantities to meet animal intake requirements. At that point, prior to inclusion in the experimental diets, it was decided to grind the cakes (electric Forage Crisher) with a 3 mm sieve to standardize the ingredient, better homogenize it, and make it difficult for the animals to select the ingredients. They were homogenized for 15 min in mixers and bagged to reduce oxidation and rancidification caused by the high-fat content and to ensure their quality. The inclusion of cakes in the diets was carried out until the diet reached 7% of EE. The proportion and chemical composition of the diets are presented in Table 2.

**Table 2:** Proportion of ingredients and chemical composition of experimental diets.

**Items**	**Diets** ^a^			
	**C40**	**CUP**	**TUC**	**C80**
**Ingredient, g/kg DM**				
Corn silage	400	400	400	400
Ground corn	432	62	132	407
Soybean meal	148	68	139	13
Ground soybean	-	-	-	145
Soybean oil	-	-	-	15
Cupuassu cake	-	450	-	-
Tucuma cake	-	-	309	-
Limestone	5	5	5	5
Mineral supplement^b^	15	15	15	15
**Nutrient, g/kg DM**				
Dry matter	643	644	648	641
Organic matter	939	926	938	942
Mineral matter	41	54	42	38
Crude Protein	149	148	143	147
Ether extract	41	67	66	78
Neutral detergent fiber	318	355	541	305
NDFcp	266	340	391	291
ADFcp	123	244	239	129
Lignin	30	80	52	28
Non-fibrous carbohydrates	503	381	358	544

a C40, control, without Amazonian cake and with 40 g/kg of ether extract (EE); CUP, the inclusion of cupuassu (CUP) cake and 70 g/kg of EE; TUC, the inclusion of tucuma cake and 70 g/kg of EE; and C80, control, without the addition of Amazonian cake and with 80 g/kg of EE.

b Composition of the product expressed in grams or milligrams/100 g of the supplement: calcium, 140 g; phosphorus, 65 g; magnesium, 10 g; sulfur, 12 g; sodium, 130 g; cobalt, 80 mg; iron, 1,000 mg; iodine, 60 mg; manganese, 3,000 mg; selenium, 10 mg; zinc, 5,000 mg; fluorine (maximum), 650 mg; vitamin A, 50.000 I.U; vitamin E, 312 I.U. NDFcp, NDF corrected for ash and protein; ADFcp, corrected for ash and protein; Non-fibrous carbohydrates, 100-(Ash+CP+EE+NDFcp).

### 2.3. Determination of intake and apparent nutrient digestibility

Daily, across 55 days, before offering the morning diet, leftovers were collected and weighed to determine the DMI (based on the difference in weight between the feed offered and rejected) and to adjust the ration to allow 10% leftovers. Animals were fed with a mixed total diet twice a day: 8:00 a.m. (60% of the diet) and 5:00 p.m. (40% of the diet).

The digestibility coefficients (DC) of DM, organic matter (OM), ash, crude protein (CP), EE, NDF, acid detergent fiber (ADF), lignin (LIG), and non-fibrous carbohydrates (CNF) were calculated using the following equation:


$$\begin{array}{l} \text{DC = }[(\text{Amount ingested-amount excreted})\text{/}\\ \;(\text{amount ingested})]\times \text{100} \end{array}$$

For this, the ingredients, offered diets, rejected portion, and feces were sampled for 5 consecutive days (days 50, 51, 52, 53, and 54). To estimate fecal production, total collection and weighing of feces were performed and stored in plastic trays, coupled to metabolic cages. All sampled materials were properly packed in plastic bags, identified, and frozen at −18°C.

### 2.4. Determination of serum metabolites

On the 55th day of the experiment, blood samples (15 ml) were collected approximately 4 h after morning feeding by puncture from the jugular vein and stored in vacuum tubes with an anticoagulant [ethylenediaminetetraacetic acid (EDTA)]. For blood fractionation, the samples were immediately centrifuged at 5,000 revolutions per minute (rpm) for 15 min. Then, the plasma was removed using a pipette and stored in microtubes at −15°C. The determination of glucose, cholesterol, triglycerides, high-density lipoprotein (HDL), albumin, and total protein concentrations was performed using an automatic biochemical analyzer (Mindray BS-200E, China), using commercial kits (Bioclin Diagnósticos—Belo Horizonte, Brazil) at the Laboratory of Physiology and Animal Reproduction of the Federal University of Viçosa.

### 2.5. Evaluation of animal performance

At the beginning and end of the experimental periods, the animals were weighed in the morning using a mobile scale both before the first meal and after 16 h of solid fasting. Total weight gain (TWG) was calculated by the difference between the final live weight (FLW), the initial live weight (ILW); The daily weight gain (DWG) was obtained by dividing the TWG by the total confinement days (70 days); and daily feed efficiency (FE), calculated by dividing the DWG by daily DMI.

### 2.6. Measurement of enteric CH_4_

Methane emitted by animals was measured *in vivo*, using the sulfur hexafluoride (SF_6_) technique (24), adapted by Primavesi et al. (25), for tropical conditions, and by Meister et al. (26), for small ruminants. SF_6_ capsules and capillary tubes used in this research were prepared and calibrated at Embrapa Southeast Livestock (São Carlos–SP). After receiving the material, the calibration was again investigated, through the weekly weighing of the capsules and flow tests of the halters. The average release rate of capsules was 2.36 ± 0.173 mg SF_6_/day.

The capsules were orally administered to animals over the adaptation period. In the period of gas collection, which occurred simultaneously with feed intake and digestibility, the eructed gases were stored in polyvinyl chloride (PVC) pipes fixed in the cages during 24-h cycles, for 5 consecutive days. At each end of the cycle, the PVC pipe hose was disconnected and its internal pressure was measured with a digital manometer for calculation purposes. The same order of collection and time was followed daily. Two ambient air samples were collected daily to determine the environmental concentration of both CH_4_ and SF_6_ and to deduct it from the animal samples.

Every two collection days, the PVC pipes were sent to the Laboratory of Sustainable Systems Analysis—LASS, at Embrapa Eastern Amazon, Belém, Pará, to determine the concentrations of CH_4_ and SF_6_, *via* gas chromatography (equipment–Wilmington, Delaware, USA), equipped with a flame ionization detector (FID), a megabore column (0.53 μm, 30 m) HP-Al/M plot (for CH_4_), an electron capture detector (μ-ECD), an HP-MolSiv megabore column (for SF_6_), and programming with temperature control. The calibration of the equipment was done with standard gases (White Martins/Praxair), with known concentrations of CH_4_ (5 ± 0.25, 10 ± 0.16, and 19 ± 0.65 ppm) and SF_6_ (34 ± 9.0, 91 ± 9.0, and 978 ± 98.0 ppt), according to Westberg et al. (27).

To identify possible gas loss from leakage caused by the translocation of the PVC pipes from one place to another, the pressure was again measured after arrival in the laboratory. The samples were then diluted by pressurization of pure nitrogen (N_2_) gas. As the injection of gases into the chromatograph could not be done directly (PVC pipe-chromatograph), there was the need to transfer the gases from the PVC pipes to small glass vials, sealed with rubber septa. From these, three subsamples (three vials) of gases were collected through an ultrafine syringe and needle and manually inserted into the equipment to determine the concentration of CH_4_ and SF_6_ gases.

The CH_4_ emission rate was calculated according to the formula: QCH_4_ = QSF_6_ × [CH_4_]/[SF_6_], where QCH_4_ = emission rate in L/h; QSF_6_ = known release rate of SF_6_ from the permeation tube; and CH_4_ and SF_6_ are the concentrations measured in the PVC pipe. From the determination of CH_4_ emissions (gram/animal/day), the emission in liters/day was calculated; grams per kilogram of DM consumed, in grams per kilogram of live weight and per daily gain (DG) (24).

### 2.7. Chemical composition

The samples of the 5 days of collection of each animal were thawed, combined in a pool of samples, and dried in a forced ventilation oven (55°C), for 72 h. After drying, they were ground in a Willey mill, sieved with a 1-mm sieve, and analyzed to determine DM (method 934.01), OM (method 924.05), ash (method 942.05), CP (method 920.87), and EE (method 920.85) using the methods by AOAC (28). NDF, FDA, and LIG were determined according to the instructions provided in a previous study (29), with the use of the ANKOM system, using α-amylase in the NDF analysis and making corrections for ash and protein (NDF and ADFcp).

### 2.8. Statistical analysis

Data analyses were performed using SAS 9.1.3 (2009), with preliminary analysis of normality (Shapiro–Wilk test) and homogeneity of variances (maximum *F*-test). The analysis of variance was performed by the PROC GLM procedure and *post-hoc* tests (Tukey–Kramer), considering the following statistical model *yij* = μ + *ti* + *eij*, where *yij* is the observed value for the response variable obtained for the *i*th treatment in its *j*th repetition, μ is the general mean, *ti* is the effect of treatment *i*, and *eij* is the experimental error associated with the observed value *yij*. The critical level of significance adopted for all analyses was a *p*-value of ≤ 0.05.

## 3. Results

### 3.1. Intake and digestibility

The intake of DM (*p* = 0.06) and OM (*p* = 0.08) was not affected by the inclusion of CUP and TUC cakes; however, lower intake was observed in animals that received TUC cake (758 g/kg DM). The CP intake of animals that received CUP (133 g/kg DM) was similar to animals fed C40 (140 g/kg DM), but animals fed the TUC diet had the lowest intake (83 g/kg DM) (*p* < 0.05). There was a higher intake of NDF by animals that received the Amazonian cakes (*p* < 0.05), especially TUC, which had 491 g/kg of DM, with the lowest intake verified in C80 (283 g/kg DM) (Table 3).

**Table 3:** Effect of the inclusion of Amazonian cakes on the intake and digestibility of nutrients of confined sheep.

**Items**	**Diets** ^**1**^				**SEM**	***P*** **-value**
	**C40**	**CUP**	**TUC**	**C80**		
**Intake, g/kg DM**						
Dry matter	1,065	1,044	758	934	0.04	0.06
Organic matter	1,027	994	753	905	0.03	0.08
Crude protein	150^a^	133^ab^	83^c^	102^bc^	0.00	< 0.01
Ether extract	48^c^	73^ab^	55^bc^	83^a^	0.00	< 0.01
Neutral detergent fiber	315^bc^	372^b^	491^a^	283^c^	0.00	< 0.01
**Digestibility, g/kg DM**						
Dry matter	732^a^	619^b^	643^b^	669^b^	7.71	< 0.01
Crude protein	743^a^	603^b^	621^b^	663^b^	11.56	< 0.01
Ether extract	929	908	928	931	5.94	0.42
Neutral detergent fiber	493^ab^	279^c^	590^a^	414^b^	58.76	< 0.01

^1^C40, control, without Amazonian cake and with 40 g/kg of ether extract (EE); CUP, the inclusion of CUP cake and 70 g/kg of EE; TUC, the inclusion of tucuma cake and 70 g/kg of EE; and C80, control, without the addition of Amazonian cake and with 80 g/kg of EE.

a − c Averages on the same line, with different letters, differ from each other (Tukey test, *p* < 0.05); SEM, standard error mean.

The digestibility of both DM (732 g/kg) and CP (743 g/kg DM) was higher in those animals that received the C40 diet (*p* < 0.05), and the digestibility of EE was not affected (*p* = 0.42). The digestibility of NDF (*p* < 0.01) was higher in sheep fed the TUC diet (590 g/kg DM) and lower in those who received CUP cakes (279 g/kg DM) (Table 3).

### 3.2. Concentration of serum metabolites

Serum glucose levels were 12.5% lower in diets with Amazonian cakes, with a difference of 8.24 mg/dl between the means (*p* = 0.03). Cholesterol levels were equal in CUP (74.46 mg/dl), TUC (76.50 mg/dl), and C80 (74.53 mg/dl) but higher than the level in C40 (57.48 mg/dl) (*p* = 0.02). The highest triglyceride level (*p* ≤ 0.01) was found on the CUP diet (27.75 mg/dl) and the lowest on the C40 diet (7.89 mg/dl). HDL was equal in the CUP (53.16 mg/dl), TUC (54.20 mg/dl), and C80 (52.7 mg/dl) diets but higher than that that in C40 (37.12 mg/dl) (*p* < 0.01). No effects were observed with regard to protein and albumin serum (*p* > 0.05).

### 3.3. Performance

Dry matter intake, considering all collections of the experimental period, was influenced by the inclusion of Amazonian cakes (*p* < 0.01). DM intake was the same for C40 (1,171 g/day), CUP (1,082 g/day), and C80 (1,065 g/day), with an average of 1,106 g/day, but was lower for TUC (750 g/day).

Although the FLW of animals was not influenced by the inclusion of Amazonian cakes (*p* = 0.06), other performance measures were influenced by their inclusion. The TWG (*p* < 0.05) was higher in the diets without the inclusion of cakes: C80 (8.16 kg) and C40 (7.8 kg). Among the treatments with the inclusion of cakes, the TWG of CUP (5.02) surpassed that of TUC (2.49 kg).

The same behavior was observed in the average DG (*p* < 0.01), with the highest value obtained among the control diets, C80 (148 g) and C40 (142 g), and among the diets that contained CUP cake (91 g), all of which exceeded the value obtained among the diets that contained TUC (45 g). The efficiency was higher for C40 and C80 diets (119 and 137), and for CUP and TUC, the means were 84 and 60, respectively (*p* < 0.01).

### 3.4. CH_4_ emissions

Methane emissions (*p* < 0.05) were higher in the C40 diet (25 g/day), the same for CUP (19 g/day) and C80 (22 g/day), and lowest in TUC treatment (15 g/day). This reduction was equivalent to 40% and was repeated in the emission data in L/day. There was no effect on CH_4_ emission in grams/kilograms of CMS day (*p* = 0.40) or in grams/kilograms of body weight (BW) (*p* = 0.06). However, the inclusion of TUC in the diet provided greater emissions (353) when the variable gram of CH_4_ per ADG was evaluated and the treatments C40 (183), CUP(221), and C80 (157) were similar (*p* < 0.01) (Table 4).

**Table 4:** Methane emissions in confined sheep with diets containing Amazonian cakes (CUP and TUC).

**Methane**	**Diets**1				**SEM**	***P*** **-value**
	**C40**	**CUP**	**TUC**	**C80**		
g/day	25^a^	19^ab^	15^b^	22^ab^	26.4	0.02
L/day	35^a^	26^ab^	21^b^	30^ab^	51.4	0.02
g/kg DMI day	24	18	21	27	60.7	0.40
g/kg BW	565	461	383	499	0.01	0.06
g/BWG day	183^b^	221^b^	353^a^	157^b^	61.4	< 0.01

^1^C40, control, without Amazonian cake and with 40 g/kg of ether extract (EE); CUP, the inclusion of cupuassu cake and 70 g/kg of EE; TUC, the inclusion of tucuma cake and 70 g/kg of EE; and C80, control, without the addition of Amazonian cake and with 80 g/kg of EE;

ab Averages on the same line, with different letters, differ from each other (*Tukey-test, p* < 0.05); SEM, standard error mean; DMI, dry matter intake; BW, body live weight.

## 4. Discussion

### 4.1. Intake and digestibility

Among the factors that can influence DMI in ruminants are acceptability, diet digestibility or fiber composition, and EE content (30, 31). Despite the variation of diets in ADF, LIG, EE and digestibility values, the overall consumption was not influenced (Tables 2, 3).

The replacement of corn and wheat bran by cupuassu cake, at up to 400 g/kg, was evaluated, and there was no effect on the sheep's consumption, which had an average of 1,800 g/day in animals weighing 25 kg (32). Increased consumption was also reported with the inclusion of CUP cake (20). In the present study, 450 g of CUP was included in the diet and no negative effects on intake were observed. The effect of the inclusion of TUC cake on consumption in sheep has not yet been reported in the literature.

Reduction in the digestibility of DM in the CUP, TUC, and C80 treatments can be explained by the EE levels in these diets (67, 66, and 78 g/kg DM), which may cause the coating effect of food particles in the rumen and/or toxic effects to rumen microorganisms (33, 34). The reduced ability of rumen microorganisms to convert unsaturated fatty acids present in the diet to saturated fatty acids, leading to their accumulation, may be another possible explanation (35).

In addition to the fat factor, diets containing Amazonian cakes had higher fiber (NDF and ADF) and LIG contents. The LIG content of the CUP diet, which was 2.5 times higher than that of control diets (C40 and C80), may have contributed to lower NDF digestibility among all treatments. However, NDF digestibility in the TUC diets was the highest despite having a higher LIG content compared to C80. This fact may als be related to the toxicity of fats and the fatty acid profile of the treatments, as the TUC cake had a higher content of saturated fatty acids compared to the CUP cake and ground soybean (36). The degree of saturation is one of the properties of lipids that determines their antimicrobial effects in the rumen and component digestibility (37, 38).

### 4.2. Concentration of serum metabolites

Assessment of the concentrations of blood metabolites helps us to understand the nutritional and metabolic status of animals (39). Furthermore, serum glucose is an important indicator of the energy status of ruminants (40). Fast fermenting carbohydrates, the main energy sources in the diet, were lower in diets with a higher percentage of fat and had a higher presence of fiber in CUP and TUC. This might have been reflected in glucose concentrations, which were higher in control diets. Another possibility is related to a probable increase in propionic acid in the rumen or glycogenic amino acids in the diet, as ruminants are neoglucogenic animals (41), and the volume of glucose in the blood is almost equivalent to the production of propionate in the rumen, which in turn is regulated by the energy source in the diet (42, 43); however, the results found are within the reference values. The increase in serum concentrations of cholesterol, triglycerides, and HDL was the result of increased lipid absorption in the intestine and increased metabolites in the bloodstream, due to the fat content of the CUP, TUC, and C80 diets. Stearic (C18:0) and linoleic acids (C18:2 n-6) present in Amazonian cakes are associated with an increase in total cholesterol levels (44). Diets supplemented with fat increase the plasma and follicular levels of cholesterol, which is related to HDL levels (45, 46).

Despite being influenced by the inclusion of cakes in the diets, blood metabolite concentrations were within the reference values for most of the variables evaluated, with the exception of triglyceride and HDL contents, in animals fed C40, which were below normal (14–46 mg/dl) and albumin, in animals of all treatments, with values slightly above the expected maximum (3.0 mg/dl) (Table 5).

**Table 5:** Blood metabolites of confined sheep with diets containing Amazonian cakes (CUP and TUC).

**Items (mg/dl)**	**RV**	**Diets** ^**1**^				**SEM**	***P*** **-value**
		**C40**	**CUP**	**TUC**	**C80**		
Glucose	50–80^2^	66.15^a^	57.97^b^	58.31^b^	66.6^a^	33.13	0.03
Total cholesterol	64–103^3^	57.48^b^	74.46^a^	76.50^a^	74.53^a^	113.17	0.02
Triglycerides	14–46^3^	7.89^c^	27.75^a^	18.61^b^	21.87^ab^	31.51	< 0.01
HDL	38–72^3^	37.12^b^	53.16^a^	54.20^a^	52.7^a^	60.54	< 0.01
Protein	6.0–7.9^2^	5.68	5.56	5.41	5.47	0.10	0.52
Albumin	2.4–3.0^2^	3.34	3.14	3.19	3.28	0.02	0.06

^1^C40, control, without Amazonian cake and with 40 g/kg of ether extract (EE); CUP, the inclusion of cupuassu cake and 70 g/kg of EE; TUC, the inclusion of tucuma cake and 70 g/kg of EE; and C80, control, without the addition of Amazonian cake and with 80 g/kg of EE;

a − c Averages on the same line, with different letters, differ from each other (*t-*student, *p* < 0.05); SEM, standard error mean. RV, reference values according to ^2^Kaneko et al., (47); ^3^Khaki et al. (48) (sheep 9 months; 50 kg); HDL, high-density lipoprotein.

Comparison of the biochemical profile of production animals between different studies is subjective, as factors such as age, sex, diet, race, body score, and the technique used to determine serum concentrations directly influence the results obtained (49, 50). The reduction in protein concentrations allowed us to evaluate the protein nutritional status of sheep fed the experimental diets and infer that the same, despite being formulated to meet the requirements of the animals, under the conditions studied, were insufficient to provide the desired weight gain.

### 4.3. Performance

The lowest averages observed in the variables such as TWG, DG, and FE, which could be attributed to animals fed diets containing CUP and TUC cakes, may occur due to the reduced DM digestibility in these treatments, which was due to the nutritional characteristics of these ingredients at the inclusion levels tested in this study. The inclusion of 309 g of TUC cake, compared to the inclusion of 450 g of CUP cake, decreased digestibility and animal performance and allowed an average gain of only 45 vs. 91 g/day for animals fed CUP cake. Animal performance is strictly dependent on DMI and digestibility, as the nutrients available in the diet will meet maintenance and production requirements (51). However, the best-performing C80 diet had lower DMI and DM digestibility, similar to TUC and CUP diets. Thus, the amino acid profile of the protein may play a role as the C80 diet has a higher protein content from soy (52). Protein quality is indicated by the amino acid composition entering the small intestine, and amino acid composition is directly linked to microbial protein formation and ruminant performance (53). Due to its quality (high protein content and essential amino acids), soybean meal is typically the main source of protein in ruminant diets (54). When the basic requirements for gain are not met, a reduction in performance occurs.

Feed efficiency can significantly reduce costs, minimize environmental impact, optimize land and resource use efficiency, and improve the overall profitability of the livestock industry (55). This reflects how much of the diet was converted into the final product, in this case, meat (56). However, by reflecting the intake and digestibility of the diet, the inclusion of Amazonian cakes in the diet decreased the FE of the animals (57).

### 4.4. CH_4_ emissions

The addition of fat to the diet increases energy content and, more recently, has been studied for CH_4_ mitigation due to the toxic effects on methanogenic ruminal microorganisms, increased carbon capture, and hydrogen sequestration for biohydrogenation (58–60). Fat causes a decrease in the population of methanogenic ruminal protozoa and an increase in biohydrogenation, serving as a sink for metabolic hydrogen and reducing its availability for the formation of CH_4_ (5, 61, 62). However, the effectiveness of this inclusion in CH_4_ reduction varies depending on the fatty acid profile of the ingredient, which makes studies with unconventional ingredients even more important (5, 63).

The inclusion of Amazonian cakes had no effect on CH_4_ emissions grams/kilograms DMI day and grams/kilograms BW, probably due to the fatty acid composition (saturated and unsaturated) of the fat present in the cake. However, CH_4_ emissions (grams/day) and (liters/day) from animals that received TUC were reduced, possibly due to reduced DMI as well as the composition of fatty acids (Table 6). For many years, the chemical constitution of the diet was used as proof of its quality. More recently, the quantification of enteric CH_4_ emissions appears as an important indicator for the evaluation of diet quality, as it is also related to FE, the reduction of energy losses, and the preservation of the environment (34, 64).

**Table 6:** DMI and performance of confined sheep with diets containing Amazonian cakes (CUP and TUC).

**Items**	**Diets** ^**1**^				**SEM**	***P*** **-value**
	**C40**	**CUP**	**TUC**	**C80**		
**Intake**						
DMI, g day	1171^a^	1082^a^	750^b^	1065^a^	0.02	< 0.01
DMI, % BW	2.7^a^	2.6^a^	1.9^b^	2.4^a^	0.05	< 0.01
**Performance**						
Initial body weight, kg	35.9	36.2	36.6	35.4	5.23	0.87
Final body weight, kg	43.7	41.2	39.1	43.6	10.26	0.06
Total weight gain, kg	7.8^ab^	5.02^bc^	2.49^c^	8.16^a^	3.17	< 0.01
Daily gain (DG), g	142^ab^	91^bc^	45^c^	148^a^	0.00	< 0.01
Feed efficiency, g gain/g DMI	119^a^	84^b^	60^b^	137^a^	0.00	< 0.01

^1^C40, control, without Amazonian cake and with 40 g/kg of ether extract (EE); CUP, the inclusion of cupuassu cake and 70 g/kg of EE; TUC, the inclusion of tucuma cake and 70 g/kg of EE; and C80, control, without the addition of Amazonian cake and with 80 g/kg of EE;

a − c Averages on the same line, with different letters, differ from each other (*Tukey-test, p* < 0.05); SEM, standard error mean; DMI, dry matter intake; BW, body live weight.

Animals fed CUP cake showed CH_4_ emissions (grams/day) similar to control treatments, suggesting that the inclusion of this ingredient did not reduce emissions (grams/day); however, it did not contribute to an increase in emissions, which could be evaluated as positive for the selection of unconventional ingredients like corn and soybeans. The highest average CH_4_ emissions in grams/DG/day were observed in TUC-treated sheep, suggesting that animals in this group were less efficient in using the diet and emitted more CH_4_ for meat production.

On the other hand, Amazonian cakes can be adapted to supplement the diet of pasture lambs. Supplementation with concentrate has already been shown to reduce CH_4_ emissions compared to an extensive system due to changes associated with rumen fermentation (5, 65).

## 5. Conclusion

The supplementation with cakes did not improve intake, digestibility and performance, did not compromise blood metabolites and did not reduce the enteric CH4 emission in confined sheep in the Amazon. However, the use of CUP cake did not contribute to an increase in CH_4_ emissions, while the inclusion of the TUC cake did. Further research should be carried out to verify these results and improve the use of these cakes in sheep feed.

## Data availability statement

The original contributions presented in the study are included in the article/supplementary material, further inquiries can be directed to the corresponding author.

## Ethics statement

The animal study was reviewed and approved by Ethics and Animal Welfare Committee of the Federal University of Pará, Faculty of Veterinary Medicine—Campus Castanhal, protocol number 8694141217.

## Author contributions

Experimental design: AS, JL-J, and BN. Experimental performance: JB, VC, and SS. Data curation: ABa, LSR, ABe, and JL-J. Formal analysis: LNR, EM, AS, and ABa. Writing-original draft: JB, TR, AS, and JL-J. All authors edited and approved the final manuscript.
